# MUC13-Associated Molecular Interactome in Pancreatic Cancer

**DOI:** 10.34133/csbj.0056

**Published:** 2026-04-22

**Authors:** Anupam Dhasmana, Swati Dhasmana, Rajasekhar Baru, Abigail Gomez, Shafiul Haque, Sheema Khan, Murali M. Yallapu, Subhash C. Chauhan

**Affiliations:** ^1^ Division of Immunology and Microbiology, Department of Medicine and Oncology, ISU, School of Medicine, University of Texas Rio Grande Valley, McAllen, TX, USA.; ^2^ South Texas Center of Excellence in Cancer Research, School of Medicine, University of Texas Rio Grande Valley, McAllen, TX, USA.; ^3^ Department of Nursing, College of Nursing and Health Sciences, Jazan University, Jazan, Saudi Arabia.; ^4^ School of Medicine, Universidad Espiritu Santo, Samborondon 091952, Ecuador.

## Abstract

Mucin-13 (MUC13) has emerged as a critical molecular player involved in tumorigenesis, cancer progression, metastasis, and evasion of immune surveillance, including in pancreatic cancer. In this report, we identified MUC13 molecular interactome using an integrated immunoprecipitation and liquid chromatography–mass spectrometry (accession no. of protein data JPST004189) approach combined with systems network biology to determine its plausible functional protein–protein interaction network and associated oncogenic mechanisms. This study reveals a total of 54 key MUC13 interactor proteins (including cyclin-dependent kinase 1, catenin delta 1, Caludin-1, ErbB3-binding protein 1, basic transcription factor 3, tight junction protein 1, etc.). Interestingly, the majority of these interacting proteins is associated with oncogenic and immune checkpoint pathways. These proteins participate in the regulation of programmed cell death protein 1/programmed cell death ligand 1, T cell receptor signaling, and posttranslational protein processing and modifications in the endoplasmic reticulum. In addition, this study suggests that MUC13 may be involved in other cellular processes such as apoptosis, autophagy, senescence, calcium signaling, microRNA activity, lysosomal function, and diabetic cardiomyopathy. Taken together, this study provides the first comprehensive elucidation of the MUC13-associated molecular interactome, which may contribute to pancreatic cancer progression and metastasis. These findings may facilitate the development of new strategies to inhibit pancreatic cancer progression via disrupting MUC13-associated oncogenic molecular network.

## Highlights

1. MUC13 is an important biomarker that appears in only a few types of cancer and is particularly notable for its strong specificity to pancreatic ductal adenocarcinoma (PDAC), with only minimal or negligible expression under normal physiological conditions.
2. Proteins such as MUC13 never work in isolation; they are work horse and participate in cellular functions in the form of interactome.
3. This study is the first comprehensive IP-LC-MS-based mapping of the MUC13 interactome.
4. MUC13-associated molecular interactome involved in various oncogenic and immune checkpoint pathways, which might initiate pancreatic cancer progression and metastasis.

## Graphical Abstract

**Figure:**
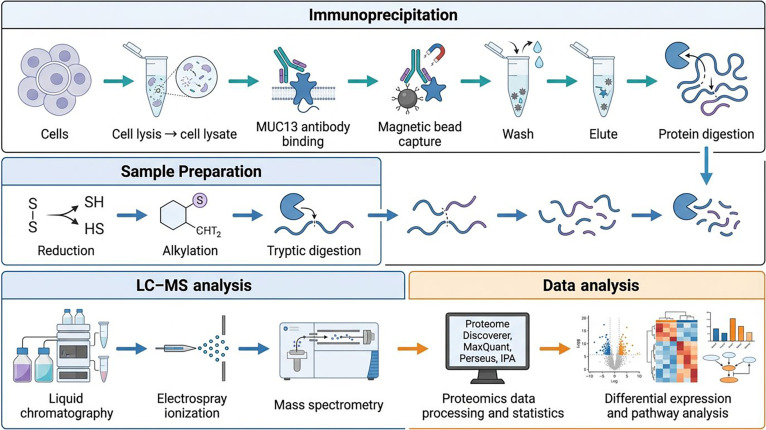

## Introduction

Pancreatic cancer is one of the most aggressive and devastating malignancies worldwide [1,2]. The median survival rate is less than 6 months, and a 5-year overall survival rate remains below 8% [3,4]. Notably, the incidence and mortality rate are nearly identical [5]. According to American Cancer Society, an estimated 67,440 new cases and 51,980 deaths are projected in year 2025. The incidence of pancreatic cancer continues to rise by approximately 1% annually [6]. The lack of effective early detection methods, fast metastatic spread, tedious surgical interventions, and chemotherapy resistance makes pancreatic cancer particularly difficult to manage [7,8]. Therefore, advancing our molecular understanding of the disease is essential for improving clinical outcomes. Such insights may also facilitate the development of new biomarkers that can either serve alone or complement the efficacy of existing biomarkers such as mucin-1 (MUC1), MUC13, carcinoembryonic antigen (CEA), and carbohydrate antigen 19-9 (CA19-9) [1].

Recently, our laboratory has identified MUC13 as a promising mucin, that is highly overexpressed in pancreatic cancer. Compared to other biomarkers, MUC13 demonstrates a higher degree of specificity for pancreatic tumors. It is a high-molecular-weight transmembrane glycoprotein characterized by a tandem repeat (TR) domain—a hallmark feature of mucins. The TR domain contains numerous serine and threonine residues, which serve as glycosylation sites. In addition, MUC13 possesses 3 epidermal growth factor (EGF)-like domains and a unique, functionally versatile cytoplasmic tail. This tail harbors potential phosphorylation sites that may regulate key cellular signaling pathways and lead further downstream protein–protein interactions [9]. Proteins are often referred as the “workhorse” of the cell; however, they rarely work alone; rather, they usually interact with other proteins within dynamic networks that direct cellular activities [10]. Every protein participates in a broad and fluctuating interactome, and the integrity of entire proteome is essential for maintaining cellular homeostasis [11]. Previous studies have suggested that MUC13 can interact with several proteins to regulate cellular activities and its overexpression contributes to enhanced tumorigenic and metastatic behavior in pancreatic cancer cells. These aggressive traits are partly mediated through physical interaction with HER2/Neu. MUC13 also promotes pancreatic cancer progression by influencing tumor glucose metabolism, thereby supporting increased proliferation, invasion, and survival [12–14].

However, a important gap remains in identifying both the direct and indirect interactors of MUC13. As proteins function cooperatively, understanding the interaction network of MUC13 is crucial. The identification of precise interactors of any protein is a highly complex process, often requiring the identification of each partner individually. Immunoprecipitation (IP) method is a widely used method for studying protein–protein interactions [15], but it is generally limited to detecting interactions among known proteins. In this study, we developed a comprehensive strategy to identify MUC13-interacting partners to elucidate its tumorigenic and metastatic functions. To achieve this, we used proteomics a high-throughput approach frequently used to investigate cancer-related molecular mechanisms [16]. This study represents the first comprehensive IP-liquid chromatography–mass spectroscopy (LC-MS)-based mapping of the MUC13 interactome in pancreatic ductal adenocarcinoma (PDAC) and integrates IP, MS, and network system biology to reveal the complex interaction landscape of the MUC13 oncoprotein.

## Materials and Methods

### PDAC cell lines and cell culture

The commercially available PDAC cell lines were acquired from American Type Culture Collection (Manassas, VA, USA). For this study, we selected differentiation grades of PDAC cell lines, such as normal pancreatic cell lines (HPNE), well-differentiated cell lines (CAPAN2 and HPAF-II), and poorly differentiated cell lines (PANC-1 and MiaPaCa). The Dulbecco’s modified Eagle’s medium (DMEM)/F12 (catalog no. 11320033, Gibco, for HAPH-II) and RPMI 1640 (catalog no. 11875-093, Gibco, for BxPC3 and SU86.86) media were used for the cell culture. The DMEM, RPMI 1640, or DMEM/F12 media supplemented with 10% fetal bovine serum and 1% (w/v) penicillin–streptomycin were used for the cell cultivation (Gibco, Thermo Fisher Scientific, Grand Island, NY, USA) under humidified atmosphere with 5% CO_2_ at 37 °C [1,17]. The cell line authentication and routine confirmation of mycoplasma-free status are performed to ensure full transparency and compliance with good laboratory practice.

### Quantitative detection of MUC13 protein in PDAC cell lines using ELISA

To quantify the protein concentration of MUC13 in various PDAC cell lines, enzyme-linked immunosorbent assay (ELISA) was carried out using a commercial kit from Novus Biologicals (catalog no. NBP2-76698) following the manufacturer’s protocol. Protein was isolated from cell lines (HPAF-II, CAPAN2, HPNE, MiaPaCa, and PANC-1). Radioimmunoprecipitation assay (RIPA) buffer was used to lyse the cells, followed by sonication for 10 s and 30-min incubation on ice. Postincubation, the samples were centrifuged at 15,000 rpm at 4 °C for 30 min. The supernatant was collected in fresh tubes and was used as sample for ELISA. Standards were prepared by serial dilution and ranged from 10 to 0.16 ng/ml. ELISA plates were precoated with the MUC13 capture antibody. The samples (100 μl) of the standards and test samples were added to the appropriate wells and incubated for 90 min at 37 °C. Postincubation, the liquid was removed, and, immediately, 100 μl of biotinylated detection antibody was added and incubated for 60 min at 37 °C. After washing with the wash buffer, 100 μl of horseradish peroxidase conjugate was added and incubated for 30 min at 37 °C before adding the substrate, and then absorbance was recorded at 450 nm using a Varioskan plate reader.

### Confocal-microscopy-based identification of MUC13 protein expression in PDAC cell lines

For the confocal microscopy, PDAC cells were cultivated at low density on 4-chamber slides (Lab-Tek) for 24 h and processed for confocal microscopy as described in a previous article [1]. Cells were incubated with anti-MUC13 *mAb* (C18 clone) [12], followed by species-specific CY3 secondary antibodies (anti-mouse, Jackson ImmunoResearch, catalog no. 715-166-151; 1:200) and mounted in 4′,6-diamidino-2-phenylindole-containing VECTASHIELD mounting medium (Vector Laboratories, Burlingame, CA H-1200). Immunofluorescence was examined and photographed under A1R HD25 confocal microscope (Nikon Instruments Inc., Melville, NY, USA). Photomicrographs were captured in the green channel.

### LC-MS sample preparation from PDAC cell lines

EasyPep Mini MS Sample Prep Kits (catalog no. A40006) from Thermo Fisher Scientific were used for protein digestion and peptide sample preparation. The tissue culture plate was washed 3 times with ice-cold phosphate-buffered saline and 100 μl of lysis buffer. After protein concentration measurement using the Pierce BCA Protein Assay Kit, samples were processed for the reduction, alkylation, protein digestion, and peptide clean-up steps. The dried peptide samples were resuspended in 0.1% formic acid for LC-MS analysis as per the instructions in the EasyPep Mini MS Sample Prep Kit manual.

### Protein abundance quantification of MUC13 in PDAC cell lines via LC-MS

LC-MS analysis was performed using an Orbitrap Exploris 240 quadrupole–Orbitrap hybrid mass spectrometer, equipped with a nanoelectrospray ion source (Thermo Fisher Scientific, Waltham, MA). Chromatographic separation of peptides was performed by LC on a nano-high-performance LC system (Ultimate 3000, Thermo Fisher Scientific, Waltham, MA), using a nano-LC column (Acclaim PepMapC100 C18, 75 μm × 15 cm, 2 μm, 100 Å; buffer A, 0.1% [v/v] formic acid in Optima LC-MS grade water; buffer B, 0.1% [v/v] formic acid in acetonitrile). One microliter of sample (1 μg/μl) was loaded onto the column. Peptides were separated on the nano-LC column using a linear gradient for 130 min at a flow rate of 300 nl/min. The mass spectrometer was operated in positive-ion mode and data-independent acquisition (DIA) mode using isolation windows of 12 mass/charge ratio with a precursor mass range of 300 to 1,200. The orbitrap resolution was set to 120,000, and DIA scan resolution was set at 30,000.

### Integration of IP and LC-MS assay to identify the interacting partners of MUC13

The standard manual protocol of Pierce Classic Magnetic IP/Co-IP Kit (catalog no. 88804) from Thermo Fisher Scientific was used for the IP assay. In this section, 8 different experiment sets were prepared according to specific cell line and IP antibodies. Cell lysates of MUC13-positive (HPAF-II) and MUC13-negative (Panc-1) cell lines were used to incubate with MUC13 antibody to immunoprecipitate MUC13 and MUC13-associated proteins. Control antibody was used to normalize nonspecific and background proteins. Protein A/G magnetic beads were used to isolate MUC13 and its associated proteins. IP proteins and flowthrough samples were processed for LC-MS to identify the MUC13 and MUC13-associated protein interactomes. The confirmation of MUC13 IP from cell lysates was performed by classical IP and immunoblotting procedures using anti-MUC13 *mAb* (C18 clone) [12].

### Data processing and analysis

LC-MS raw data were analyzed using Proteome Discover 2.5 software with DIA analysis workflow. Phosphorylation, methylation, and carbamidomethylation were set as a chemical modification option. In cleavage reagent, trypsin was selected. Data were searched against human protein sequences using from www.uniprot.org. For processing workflow, the Processing WF_Hybrid and PWF_Hybrid_Precursor_Quan_and_LFQ_MPS_SequestHT_Percolator option were selected. For Consensus workflow, CWF_Comprehensive_Enhanced Annotation_LFQ_and_Precursor_Quan option was selected. All raw files were uploaded in the processing steps, followed by grouping and quantification steps to run the samples. Publicly available gene interaction network server [18,19] was used to create a raw interaction file of global gene network. Cytoscape was used to create an interactive network, followed by functional enrichment analysis [19]. MUC13-associated proteins and their expression status were correlated with pancreatic cancer patients’ survival using publicly available datasets [20].

## Results

### Protein abundance quantification of MUC13 protein in PDAC cell lines via LC-MS analysis suggests MUC13 expression in HPAF-II cells

**Fig. 1.**
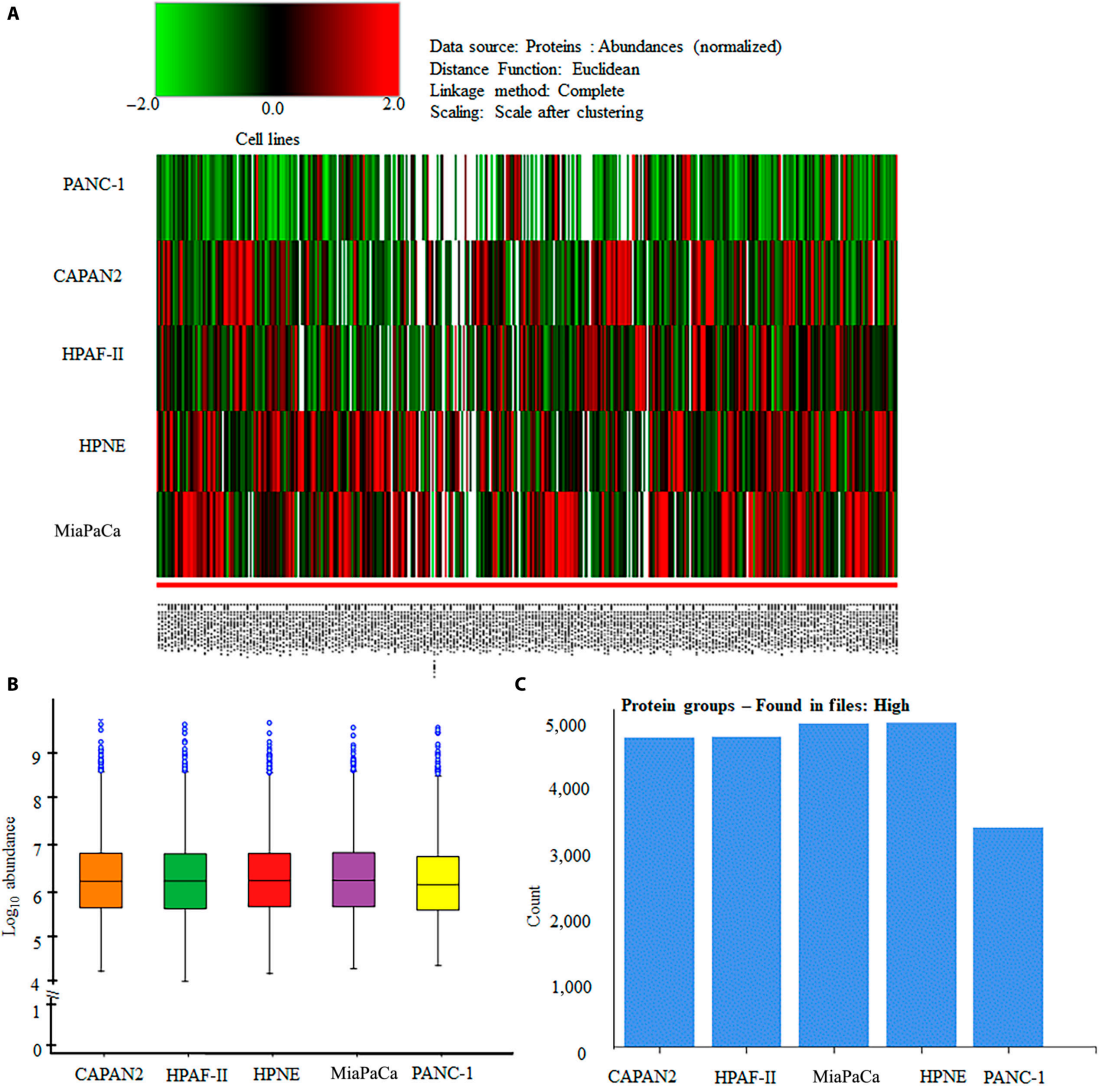
Proteomic featuring of all 5 pancreatic ductal adenocarcinoma (PDAC) cell lines. (A) Heatmap of all 5 PDAC cell lines. (B) Log_10_ abundance of all 5 PDAC cell lines, which was demonstrated the minimum and maximum values of log_10_ abundance. (C) The total number of high coverage abundant proteins was determined for each PDAC cell lines. CAPAN2 sequenced 6,412 proteins (5,142 proteins have high coverage and 1,270 proteins showed peak found); HPAF-II sequenced total 6,426 proteins (5,121 high coverage and 1,305 peak found); HPNE sequenced 6,487 proteins (5,347 high coverage and 1,140 peak found); MiaPaCa sequenced total 6,412 proteins (5,361 high coverage and 1,051 peak found); and Panc-1 sequenced total 5,617 proteins (3,635 high coverage and 1,982 peak found).

The LC-MS analysis of all 5 PDAC cell lines identified 6,412 proteins in CAPAN2, 6,426 proteins in HPAF-II, 6,412 proteins in MiaPaCa, 5,617 proteins in PANC-1, and 6,487 proteins in HPNE cells. Only high-coverage proteins were selected for further analysis. The heatmap, total log_10_ protein abundance (with the median log_10_ abundance across all cell lines ranging between 6 and 6.5), and total protein counts (ranging from 3,600 to 5,361) detected in the cell lines are presented in Fig. 1A to C.

**Table 1.** Final 54 unique interactor proteins among all proteins of HPAF-II MUC13_IP. In this table, the various features of selected proteins were mentioned, including percentage of coverage through identified peptides, total number of unique peptides, total quantitative (Quan) numbers, correlation value with mucin-13 (MUC13), and *P* value of this correlation.

Unique proteins in HPAF-II MUC1_IP	Coverage %	Unique peptides	Quan no.	*R* value correlation with MUC13	*P* value
LDHB	44	15	7.9	−0.18	0.00064
MUC13	22	8	646.7	1	0
LYZ	48	6	698.4	0.84	1.80 × 10^−94^
CLINT1	28	17	227.5	0.8	1.7 × 10^−80^
AP2B1	37	16	66.7	0.8	1.6 × 10^−80^
SLC25A24	44	20	53.4	0.8	1.5 × 10^−79^
YWHAB	51	5	43.3	0.8	3.60 × 10^−79^
ATP2A2	41	38	24.9	0.8	6.7 × 10^−78^
SNRPD3	48	4	115.3	0.79	1.3 × 10^−75^
SFXN1	39	10	43.9	0.79	4.7 × 10^−75^
MYO1D	4	3	212.8	0.78	1.6 × 10^−71^
SLIRP	69	7	34.7	0.78	4.4 × 10^−73^
CDK1	63	13	34.6	0.78	6.2 × 10^−73^
MED10	16	2	369.1	0.77	8.3 × 10^−70^
SSBP1	43	6	52.2	0.77	3.8 × 10^−70^
SERBP1	41	14	42.4	0.77	3.8 × 10^−70^
BCAP31	49	13	36.3	0.77	1.1 × 10^−70^
BTF3	55	7	26.1	0.77	1.2 × 10^−68^
MRPL30	29	4	581.8	0.76	7.2 × 10^−68^
SFXN3	48	12	31.7	0.76	7.7 × 10^−68^
HMGB2	27	4	19.3	0.76	2.2 × 10^−66^
CAND1	35	41	18.4	0.76	3.2 × 10^−68^
C1QBP	41	7	15.3	0.76	6 × 10^−67^
DNAJC17	13	4	233.3	0.75	7.8 × 10^−64^
AP2M1	40	16	122.7	0.75	7.6 × 10^−65^
PA2G4	56	19	108.1	0.75	7.1 × 10^−64^
ANXA4	57	22	35.5	0.75	4.6 × 10^−65^
CLTA	22	6	28.2	0.75	2.1 × 10^−65^
CCT6A	40	22	18.8	0.75	2.1 × 10^−63^
PSAP	35	17	22.1	0.74	3.3 × 10^−62^
PRKCSH	35	15	14.4	0.74	6 × 10^−63^
U2AF2	40	13	7.9	0.74	1.2 × 10^−62^
TJP1	35	47	156	0.73	5.8 × 10^−60^
BAIAP2L1	48	20	153.2	0.73	2 × 10^−58^
NUDT21	34	8	88.9	0.71	4.7 × 10^−54^
SUDS3	6	2	39.9	0.71	5.9 × 10^−55^
TMPO	44	11	99.7	0.7	7.4 × 10^−53^
CANX	41	28	32.1	0.69	2.8 × 10^−51^
ALDH1A3	55	20	15.2	0.69	2.9 × 10^−50^
CTNND1	50	37	143	0.68	6.8 × 10^−48^
CS	27	12	11.5	0.67	1.5 × 10^−46^
KRT79	8	1	24.4	0.65	1.9 × 10^−43^
EIF4A1	51	12	4.8	0.65	2 × 10^−43^
PDIA6	31	12	16.9	0.63	8.2 × 10^−41^
VIL1	43	30	141.7	0.61	2 × 10^−36^
SFPQ	30	22	17.4	0.61	1.8 × 10^−37^
NOS1AP	12	4	921.1	0.59	4.60 × 10^−34^
U2SURP	23	21	435.3	0.59	1.4 × 10^−34^
H1-10	36	7	68	0.53	7.7 × 10^−27^
PALM2AKAP2	18	15	384.2	0.45	1.4 × 10^−18^
RPL3	51	22	8.7	0.41	1.1 × 10^−15^
AHCY	43	18	11.4	0.37	5.3 × 10^−13^
RPSA	52	13	6.4	0.29	5.4 × 10^−08^
RPL10	37	7	7.2	0.23	1.3 × 10^−05^

**Fig. 2.**
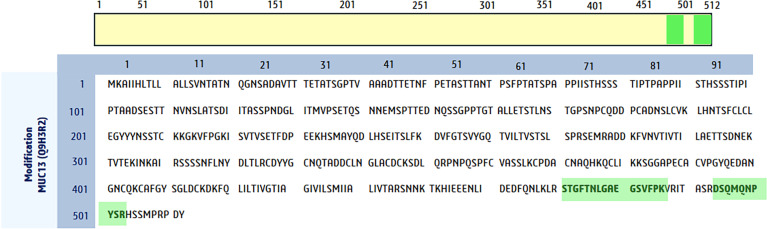
These highlighted peptide sequences of mucin-13 (MUC13) were identified as unique MUC13 peptides in the samples, and these peptides confirmed the presence of MUC13.

**Fig. 3.**
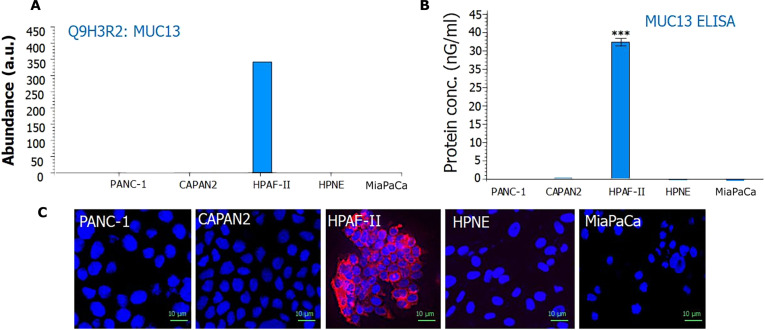
Protein expression mucin-13 (MUC13). (A) Quantitative (Quan) channel abundance of MUC13. (B) Enzyme-linked immunosorbent assay (ELISA) of MUC13 in various cell lines. (C) Confocal microscopy of MUC13 in various cell lines. HPAF-II was showing high expression of MUC13.

The presence of MUC13 was markedly higher in HPAF-II cells, whereas the other cell lines showed very little or no MUC13 expression. In the HPAF-II cell line, 2 MUC13 peptides were identified by LC-MS analysis: one 16-mer peptide (STGFTNLGAEGSVFPK) and one 10-mer peptide (DSQMQNPYSR) (Fig. 2). These peptides were validated through a global protein BLAST search, showing 100% identity with MUC13 (Fig. S1A). Furthermore, fragment matching (Fig. S1B) and spectrum segment analyses (Fig. S1C) confirmed that the peptide sequences perfectly aligned with MUC13. Together, these findings support the presence of MUC13 in the HPAF-II cell line. In HPAF-II cells, a total of 341 peptides were identified (Fig. 3A), whereas the other cell lines did not show detectable peptide abundance (all processed data from the raw files are provided in File S1).

### Quantification and spotting of MUC13 using ELISA and confocal microscopy in PDAC cells

ELISA was performed to quantify the total concentration of MUC13 in the PDAC cell lines. Among these, HPAF-II exhibited high MUC13 expression, with concentrations of approximately 40 ng/ml, whereas CAPAN2 cells showed low expression (2.2 ng/ml). In the other cell lines (PANC-1, HPNE, and MiaPaCa), MUC13 was undetectable (Fig. 3B). A similar expression pattern was observed through confocal microscopy (Fig. 3C).

### IP-MS integration reveals MUC13-associated proteins in HPAF-II cells

**Fig. 4.**
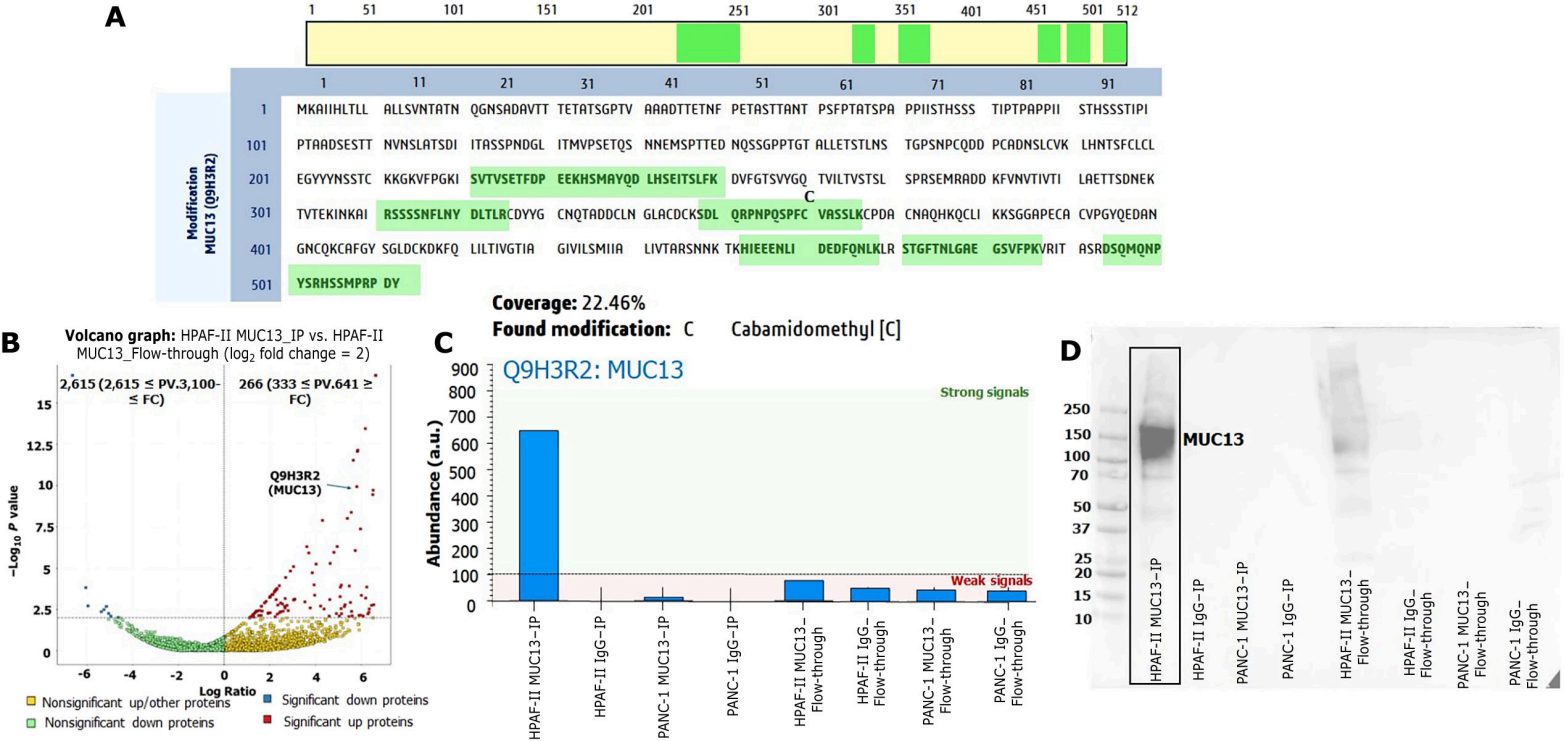
Liquid chromatography–mass spectroscopy (LC-MS) protein abundance analysis: In these experimental sets, HPAF-II MUC13_IP identified 302 proteins; HPAF-II IgG_IP identified 202 proteins; HPAF-II MUC13_Flow-through identified 4,735 proteins; HPAF-II IgG_Flow-through identified 5,110 proteins; PANC-1 MUC13_IP identified 316 proteins; PANC-1 IgG_IP identified 141 proteins; PANC-1 MUC13_Flow-through identified 4,845 proteins; and PANC-1 IgG_Flow-through identified 5,279 proteins. (A) Six unique mucin-13 (MUC13) peptides found in LC-MS. (B) Volcano graph: HPAF-II immunoprecipitation (IP) versus HPAF-II flowthrough. A total of 2,615 proteins were found down-regulated, and around 266 proteins were found up-regulated. The threshold of log_2_ fold change (FC) was selected 2. PV, *P* value. (C) Quantitative (Quan) channel abundance of MUC13 in various experimental sets. (D) Western blotting (*n* = 2) of MUC13 IP and other experimental sets demonstrated higher expression of MUC13 found in HFAF-II MUC13_IP.

IP was performed using MUC13 antibodies on MUC13-positive HPAF-II and MUC13-negative PANC-1 cell lysates, generating 8 experimental sets: HPAF-II MUC13_IP, HPAF-II IgG_IP, HPAF-II MUC13_Flow-through, HPAF-II IgG_Flow-through, PANC-1 MUC13_IP, PANC-1 IgG_IP, PANC-1 MUC13_Flow-through, and PANC-1 IgG_Flow-through. MUC13 antibodies were used as the test condition for both HPAF-II and PANC-1, while immunoglobulin G (IgG) antibodies served as a negative control. All 8 sets were digested and analyzed using the Orbitrap Exploris 240 LC-MS system. Following data processing of the IP-MS samples, only high-coverage proteins were selected from each of the 8 experimental sets. At this stage, a high abundance of MUC13 was detected in the HPAF-II samples. A total of 6 unique MUC13 peptides (SVTVSETFDPEEKHSMAYQDLHSEITSLFK, RSSSSNFLNYDLTLR, SDLQRPNPQSPFCVASSLK, HIEEENLIDEDFQNLK, STGFTNLGAEGSVFPK, and DSQMQNPYSRHSSMPRPDY) were identified in the MUC13-antibody-pulled IP samples from HPAF-II cells (Fig. 4A). None of the remaining experimental sets showed detectable levels of MUC13 peptides (all processed data from the raw files are provided in File S2).

The volcano plot was generated to identify differentially expressed proteins between HPAF-II MUC13_IP and HPAF-II MUC13_Flow-through samples. In this analysis, MUC13 was among the most significantly differentiated proteins in both datasets (Fig. 4B and File S3). The quantitative (Quan) channels also supported this trend: The HPAF-II MUC13_IP sample showed the highest MUC13 peptide abundance, with a value of approximately 647, indicating a very strong signal. In contrast, the other experimental sets exhibited very weak or negligible MUC13 signals (Fig. 4C). To cross-validate the IP-MS data, we performed the Western blot analysis on all 8 experimental sets. Consistent with the MS results, the HPAF-II MUC13_IP sample showed strong MUC13 protein expression, whereas the remaining sets showed no detectable immunoreactivity with the MUC13 antibody.

### MUC13 forms a molecular complex with ~54 proteins

**Fig. 5.**
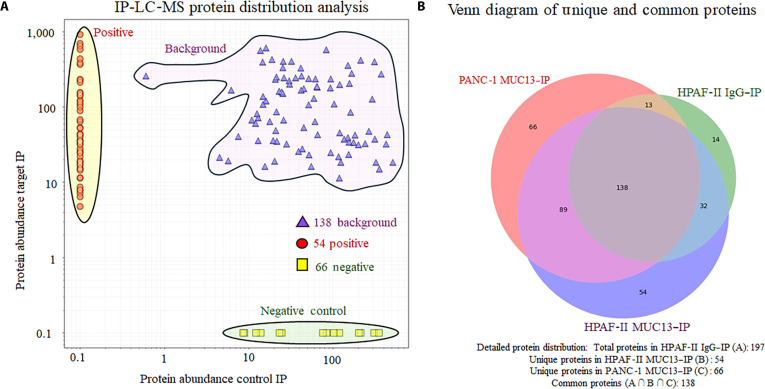
(A) Immunoprecipitation (IP)-liquid chromatography–mass spectroscopy (LC-MS) protein distribution analysis. (B) Venn diagram of unique and common proteins.

To identify the true interactors of MUC13, we performed the protein normalization using HPAF-II MUC13_IP proteins as the test group, HPAF-II IgG_IP proteins as the negative control for the MUC13 antibody, and PANC-1 MUC13_IP proteins as the negative control for the cell line. In this analysis, a total of 138 proteins that were common across all 3 experimental sets were identified and removed as background noise. In addition, 66 proteins unique to the PANC-1 MUC13_IP group were removed as cell-line-specific negative controls. Finally, 54 proteins unique to the HPAF-II MUC13_IP samples were identified and selected as potential MUC13 interactors, with a likelihood of direct or indirect interaction (Fig. 5A). These results were further visualized using a Venn diagram (Fig. 5B).

### Interactome and functional enrichment analysis

The final set of 54 proteins showed interconnectivity and formed a coherent MUC13 interactome along with its direct interactors (Fig. S2). These 54 proteins were enriched in several important pathways (false discovery rate greater than 0.05), including WNT signaling pathways, gap junction trafficking and regulation, apoptosis, major histocompatibility complex class I pathways, programmed cell death, checkpoint kinase 1/2 (Chk1/Chk2)-mediated inactivation of cyclins, Hippo signaling, and β-catenin-independent WNT signaling (File S4). Further network expansion revealed 5 additional interacting proteins forming a second-layer network, increasing the interactome from 54 to 304 proteins (File S5). The purpose of this expansion was to evaluate the broader, upstream, long-term biological impact of the MUC13-associated interactome. This extended interactome was enriched in several key pathways and disease-associated processes, including programmed cell death ligand 1 expression and programmed cell death protein 1 checkpoint signaling in cancer, acute myeloid leukemia, epithelial cell signaling in *Helicobacter pylori* infection, adherens junctions, hepatitis C, hypoxia-inducible factor-1 signaling, pancreatic cancer, T cell receptor signaling, cellular senescence, HIV-1 infection, small cell lung cancer, cell cycle regulation, apoptosis, microRNAs in cancer, lysosomal pathways, proteoglycans in cancer, human T cell leukemia virus-1 infection, viral carcinogenesis, human papillomavirus infection, and multiple cancer-related pathways (Fig. S3 and File S6).

### MUC13-associated proteins and their status in pancreatic cancer

After identifying the final 54 interactors, they were further analyzed to determine their relevance in pancreatic cancer. Of these 54 proteins, 40 interactors were selected to evaluate their potential impact on pancreatic cancer. Selection was based on their correlation with MUC13, and interactors with an *R* value greater than 0.675 were chosen for further analysis (Table 1). GEPIA was used to assess these interactors based on their significantly elevated expression levels and their association with reduced overall survival. Following this rigorous screening, a total of 23 interactors—CLINT1, LYZ, AP2B1, YWHAB, SLC25A24, MYO1D, SNRPD3, CDK1, SSBP1, BTF3, SERBP1, MRPL30, SFXNL3, HMGB2, CAND1, AP2M1, PA2G4, BAIAP2L1, TJP1, NUDT21, TMPO, CTNND1, and ALDH1A3—were identified (expression and survival data for some proteins are shown in Fig. S4).

## Discussion

MUC13 has emerged as a novel and promising oncogenic biomarker for pancreatic cancer. Although several biomarkers are currently used for PDAC diagnosis, their major limitation remains the lack of specificity. Among mucins, MUC1 has long been considered the gold standard; however, its expression in more than 25 different organs reduces its specificity and makes it a broad-spectrum marker for various malignancies. In contrast, MUC13 exhibits highly selective expression in pancreatic cancer tissues. This distinct expression pattern emphasizes MUC13’s potential as a more precise diagnostic biomarker for PDAC [14].

Previous studies consistently demonstrate that MUC13 expression is detectable from early to advanced stages of the PDAC, while its expression in normal pancreatic tissue is minimal to untraceable. Based on these findings, the current study shift focuses on elucidating the molecular interactions of MUC13 in PDAC [21–23]. To achieve this, multipronged strategy incorporating IP, MS, and systems network biology was used. This integrated approach enabled a comprehensive exploration of MUC13’s functional protein partners—collectively referred to as the “interactome”. This is the first comprehensive IP-LC-MS–based mapping of the MUC13 interactome in PDAC, revealing a probable interactor and their involvement in oncogenic, immune checkpoint, and metabolic pathways.

Although conventional IP techniques, effective for identifying protein complexes, they are time consuming and typically limited to one interaction at a time. By integrating LC-MS with IP, we gained the ability to simultaneously identify hundreds of proteins closely associated with MUC13 in PDAC cells. Initial LC-MS experiment confirmed high MUC13 peptide abundance in the HPAF-II cells; in contrast, there were no MUC13 peptide signals in MiaPaCa, CAPAN2, PANC-1, and HPNE cells. Two peptides of MUC13, 16-mer (STGFTNLGAEGSVFPK) and 10-mer (DSQMQNPYSR), were identified, both unique peptides located within in the cytoplasmic domain of MUC13. In total peptide abundance, 341 peptides of MUC13 were quantified in HPAF-II cell line.

To validate these findings, ELISA and confocal microscopy were performed, and both confirm the high MUC13 expression found in HPAF-II cells. Following this confirmation, IP was performed to pull down the MUC13 protein along with its associated interactome. Parallelly, LC-MS was integrated to identify the best possible interactors. HPAF-II and PANC-1 cells (MUC13-negative cell line) were subjected to IP using both MUC13 antibody and IgG antibody, which is used for normalization (HPAF-II MUC13_IP, HPAF-II IgG_IP, PANC-1 MUC13_IP, and PANC-1 IgG_IP). Integration of IP-MS/LC-MS revealed 6 unique peptides (SVTVSETFDPEEKHSMAYQDLHSEITSLFK, RSSSSNFLNYDLTLR, SDLQRPNPQSPFCVASSLK, HIEEENLIDEDFQNLK, STGFTNLGAEGSVFPK, and DSQMQNPYSRHSSMPRPDY) with high MUC13 coverage. These peptides originated from the SEA (sperm protein, enterokinase, and agrin domain) domain, EGF (epidermal growth factor)-like domain 2, TR (tandem repeat) domain, and cytoplasmic domain of MUC13. Importantly, these peptides were highly specific to MUC13 and contributed to approximately 22.46% of total protein coverage. Quantitative analysis further revealed 647 MUC13 peptides in the HPAF-II MUC13_IP sample, indicating successful enrichment of MUC13 compared to the initial LC-MS experiment. Immunoblotting across all experimental sets confirmed strong MUC13 expression in the HPAF-II MUC13_IP group. In this experimental set, a total of 320 MUC13-associated proteins were identified in “high” abundance. Those were normalized using PANC-1 MUC13_IP (for removal of negative control proteins) and HPAF-II IgG_IP (for removal of background noise proteins). After stringent filtration, a total of 54 probable MUC13-associated proteins (first layer) were identified. These proteins were enriched with various pathways, such as WNT pathways, gap junction trafficking and regulation, apoptosis, major histocompatibility complex class, programmed cell death, Chk1/Chk2-mediated inactivation of cyclin, Hippo signaling, and β-catenin-independent WNT signaling.

**Fig. 6.**
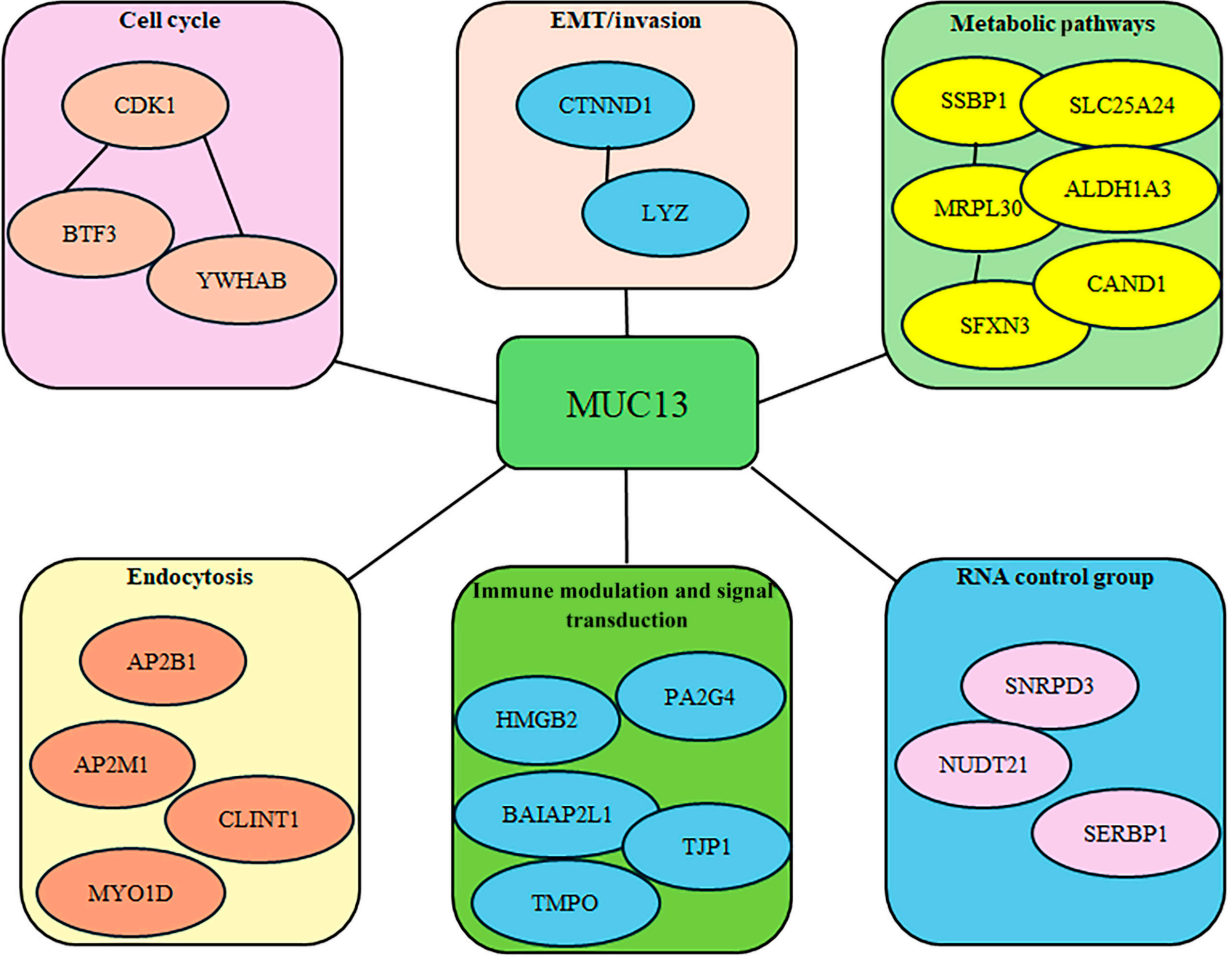
Involvement of mucin-13 (MUC13) and its associated 23 proteins in various cellular pathways. EMT, epithelial–mesenchymal transition.

The network of these 54 proteins was extended to second layer, revealing deeper involvement in multiple molecular pathways. The second layer included several key proteins such as cytotoxic T-lymphocyte-associated protein 4, TP53, EGF receptor, caspase 8, Catenin β1, polymerase delta-interacting protein 2, polymerase gamma, RELA, tumor necrosis factor, etc. Functional enrichment analysis indicated that these proteins participated in numerous cancer-related pathways including pancreatic cancer, endometrial cancer, prostate cancer, small cell lung cancer, viral carcinogenesis, microRNA in cancers, human cytomegalovirus, human papillomavirus, programmed cell death ligand 1 expression–programmed cell death protein 1 checkpoint pathways in cancer, cell cycle, apoptosis, central carbon metabolism in cancer, etc. Several noncancerous pathways were also enriched, including endocrine- and other factor-regulated pathways, calcium reabsorptions, thyroid hormone signaling, diabetic cardiomyopathy, adherens junctions, Chagas disease, bacterial invasion of epithelial cells, protein processing in endoplasmic reticulum, HIV infection, coronavirus disease, and pathways of neurodegenerative diseases. Further screening of the initial 54 probable interactome proteins revealed that 23 proteins that correlated with MUC13 had a connection to pancreatic cancer and were associated with a low overall survival rate when these genes were overexpressed in pancreatic cancer patients. This type of analysis might be a plausible direction to study the cumulative impact of all these genes, along with MUC13, on the overall survival of patients. These interactors CLINT1, SLC25A24, SSBP1, SFXN3, PA2G4, BAIAP2L1, LYZ, SNRPD3, BTF3, HMGB2, TJP1, CTNND1, AP2B1, MYO1D, SERBP1, CAND1, NUDT21, ALDH1A3, YWHAB, CDK1, MRPL30, AP2M1, and TMPO represent promising candidates for future diagnostic and prognostic biomarker development (Table 2 and Fig. 6).

**Table 2.** Mucin-13 (MUC13)-associated proteins and their functional role in cancer

S. no.	Name of protein	Function	Strength of evidence ranking basis	References
1	CLINT1	Direct PDAC evidence reported by the user summary as reducing proliferation, migration, invasion, and chemoresistance when inhibited in PDAC; ranked high but kept slightly below genes with multiple easily verifiable PubMed hits in this pass.	Strong direct PDAC functional evidence from cited source list	[24]
2	SLC25A24	Evidence appears to be mainly The Cancer Genome Atlas or cohort based in PDAC with links to mitochondrial metabolism and survival, but I did not verify a strong PDAC mechanistic paper in this pass.	Moderate indirect PDAC evidence	[25]
3	SSBP1	No direct PDAC study verified here, but there is strong evidence in other cancers that SSBP1 affects mitochondrial function, oxidative stress handling, and therapy response.	Indirect cross-cancer mechanistic evidence	[26]
4	SFXN3	No direct PDAC study confirmed here. Biologically plausible through mitochondrial serine transport and one-carbon metabolism, both relevant to cancer metabolism.	Indirect biological rationale only	[27]
5	EBP1	General oncogenic and prognostic relevance across cancers and user-stated correlation with prognosis in PDAC, but I did not verify a PDAC-specific mechanistic paper here.	Weak to moderate evidence pending exact PDAC source	[28]
6	BAIAP2L1	Direct PDAC evidence in user summary via PI3K–AKT pathway; also supported by strong oncogenic evidence in other cancers showing proliferation, invasion, poor prognosis, and chemoresistance.	Strong but partly indirect evidence	[29]
7	LYZ	No direct PDAC evidence verified here; broad cancer relevance exists but specificity for pancreatic cancer appears weak.	Weak indirect evidence	[30]
8	SNRPD3	No direct PDAC evidence verified here; strong cancer relevance in MYCN-driven neuroblastoma but not pancreatic cancer specifically.	Weak indirect evidence	[31]
9	BTF3	Direct pancreatic cancer evidence in the user summary for apoptosis and transcriptional regulation but not independently verified here; strong cancer-supportive biology exists in other tumors.	Moderate evidence	[32]
10	HMGB2	Direct PDAC evidence in user summary, though the specific PubMed hit was not verified in this pass. HMGB family biology is clearly relevant in pancreatic cancer, but HMGB2-specific ranking stays mid-high pending exact source confirmation.	Moderate direct PDAC evidence	[33]
11	TJP1	Evidence in pancreatic cancer is mainly prognostic and expression-based, with additional functional evidence from other tumors showing roles in migration, invasion, and proliferation.	Moderate direct PDAC plus supportive cross-cancer functional evidence	[34]
12	CTNND1	Direct pancreatic cancer link in the user summary through metastatic potential; placed mid-table because evidence appears specific but narrower.	Moderate direct PDAC evidence	[35]
13	AP2B1	No clear direct PDAC study verified here; recurrently appears in your pathway analysis and has supportive oncogenic evidence in other cancers.	Indirect but biologically plausible evidence	[36,37]
14	MYO1D	No direct PDAC evidence verified; cancer-related support comes from acute myeloid leukemia and other malignancies, so relevance to PDAC remains speculative.	Weak indirect evidence	[38]
15	SREBP1	Direct PDAC evidence noted by the user as promoting tumorigenesis and growth via SOX9, which is strong biologically, although the exact paper was not independently fetched in this pass.	Moderate direct PDAC evidence	[39]
16	CAND1	No direct PDAC evidence verified here; role inferred from other cancers and metabolic regulation.	Weak indirect evidence	[40]
17	NUDT21	Direct PDAC evidence; promotes pancreatic cancer pathogenesis through alternative polyadenylation and PI3K–AKT signaling, including interaction with NDUFS2 and regulation of MZT1 3′ untranslated region usage.	Very strong direct PDAC mechanistic evidence	[41,42]
18	ALDH1A3	Direct PDAC evidence; associated with poor prognosis and promotes metastasis, glycolysis, and aggressive basal-like pancreatic cancer programs.	Very strong direct PDAC mechanistic and prognostic evidence	[43]
19	YWHAB	Mostly expression or database-level support in PDAC plus broad cancer relevance; no direct mechanistic PDAC paper verified in this pass.	Indirect PDAC evidence	
20	CDK1	Strong direct PDAC evidence; highly expressed in pancreatic cancer, linked to prognosis, stemness, KRAS dependency, and therapeutic vulnerability; silencing/inhibition suppresses PDAC growth and stem-like properties.	Very strong direct PDAC mechanism and translational evidence	[44]
21	MRPL30	Primarily mitochondrial housekeeping relevance with no direct PDAC evidence verified in this pass.	Weak biological plausibility only	
22	AP2M1	No direct PDAC paper verified here; evidence comes mainly from other cancers, but pathway enrichment repeatedly implicated vesicle trafficking and internalization modules containing AP2M1.	Indirect but pathway-supported evidence	[45]
23	TMPO	Reported overexpressed across digestive tract cancers with possible roles in motility including pancreas, but PDAC-specific mechanistic depth seems limited.	Moderate to limited evidence	[46]

PDAC, pancreatic ductal adenocarcinoma; PI3K, phosphatidylinositol 3-kinase.

In-depth analysis of these proteins suggests that MUC13 and its probable interactors form a very significant interactome that may participate in important pathways of PDAC. Although some proteins remain under investigation, they may hold substantial potential as effective biomarkers in the future. A key limitation of the study is the need for validation in additional MUC13-positive models and validate the interaction with co-IP and other confirmatory assays. Notably, many of the identified interactors have not been previously characterized in pancreatic cancer, yet they demonstrate associations with tumor biology, further reinforcing the value of interactome-based research.

## Conclusion

This study highlights the essential role of MUC13 not only as a highly specific biomarker but also as a functional contributor to pancreatic cancer pathogenesis through its extensive interactome. By integrating IP, MS, and systems network biology, 54 probable MUC13-associated proteins were identified that are involved in tumor progression, immune regulation, metabolic control, and cellular signaling. Among these, 23 key interactors showed most probable correlation with MUC13 and associated with poor overall survival in pancreatic cancer, highlighting their potential as novel diagnostic and prognostic markers. These findings suggest that MUC13 is more than a surface mucin; it may be a central molecular hub protein within the PDAC cellular environment, influencing diverse oncogenic and immunomodulatory pathways.

Future functional studies are needed to confirm the mechanistic roles of these interactors, but the current data provide a foundation for developing targeted diagnostic tools and therapeutic strategies focused on MUC13 and its plausible associated protein network. Overall, this work opens new avenues for precision oncology in pancreatic cancer by leveraging the untapped potential of the MUC13 interactome.

## Data Availability

The study dataset containing raw files, peaks, and meta results is deposited in jPOST (https://repository.jpostdb.org/entry/JPST004189). The bioinformatics output and other supporting data are provided as Files S1 to S4 within the manuscript.
