# Supplementary material for: MUC13-Associated Molecular Interactome in Pancreatic Cancer
Source: Comput Struct Biotechnol J. 2026 Apr 22;35(1):0056. doi: 10.34133/csbj.0056 (PMC13100349; doi:10.34133/csbj.0056)
Supplement: Supplementary 1 — Files S1 to S6 Figs. S1 to S4 [file csbj.0056.f1.zip › Supplemantary Figures.docx]

Supplementary figures


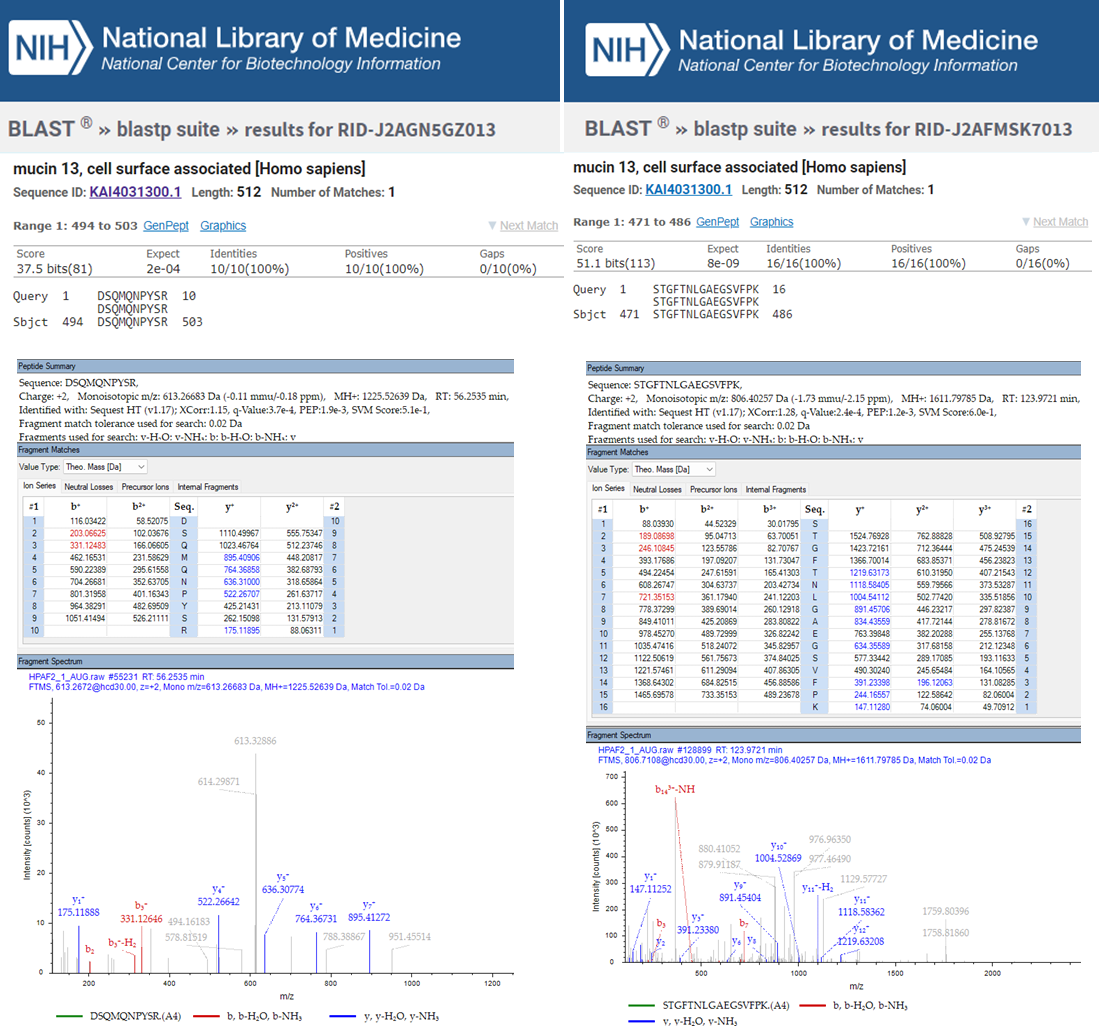


Supplementary figure 1: a) MUC13 peptide featuring among all qualified proteins. This sequence was identified two unique peptides of MUC13. b & c) BLAST analysis demonstrates the unique peptides aligned 100% with MUC13 protein sequences.


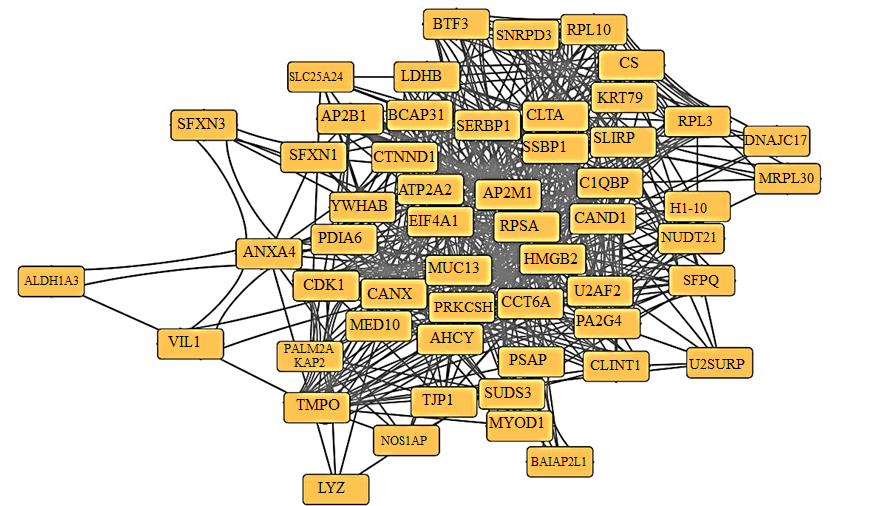


**Supplementary figure 2:** A protein-protein interactive network of all unique 54 proteins.


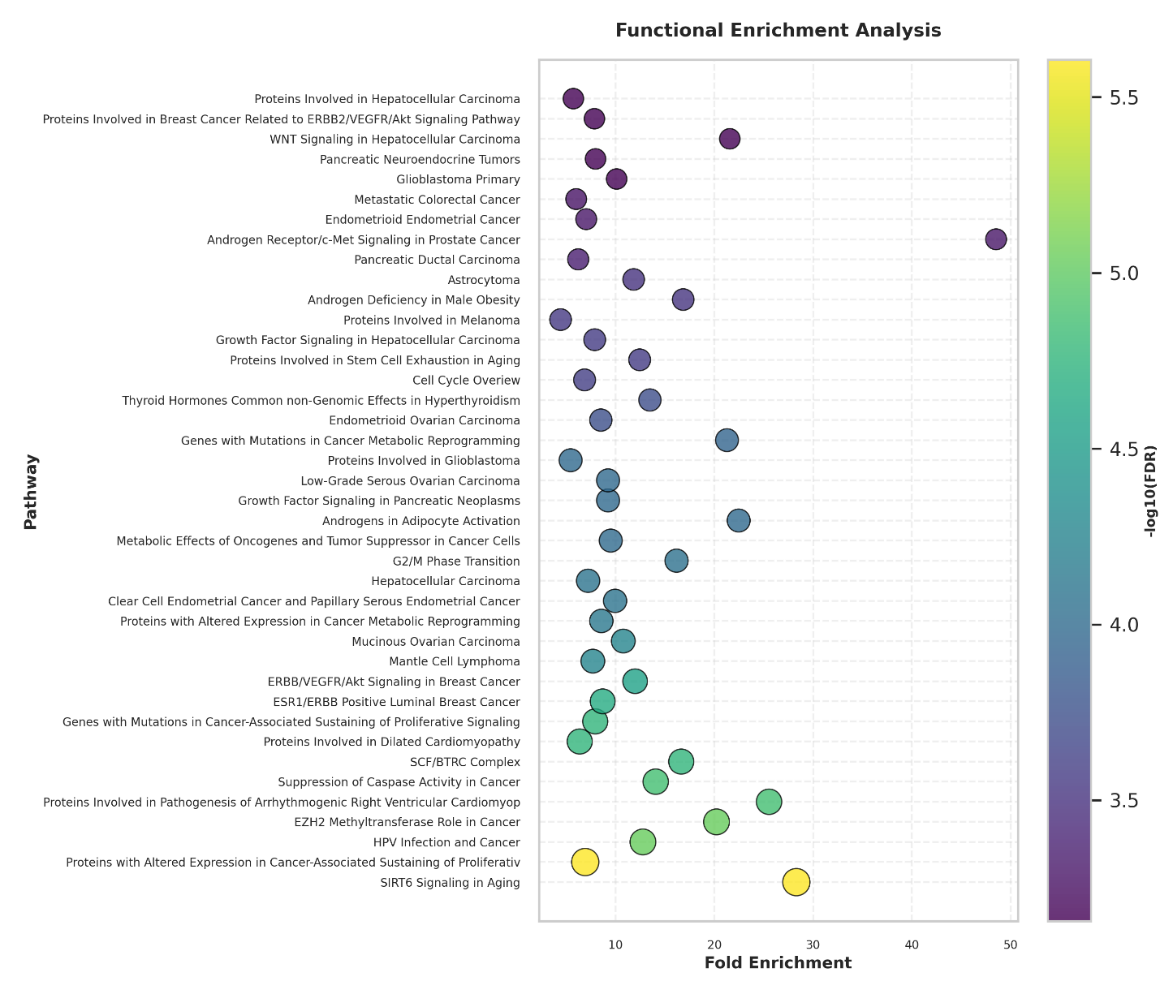


**Supplementary figure 3:** Functional enrichment analysis of all 54 proteins along with its second layering.


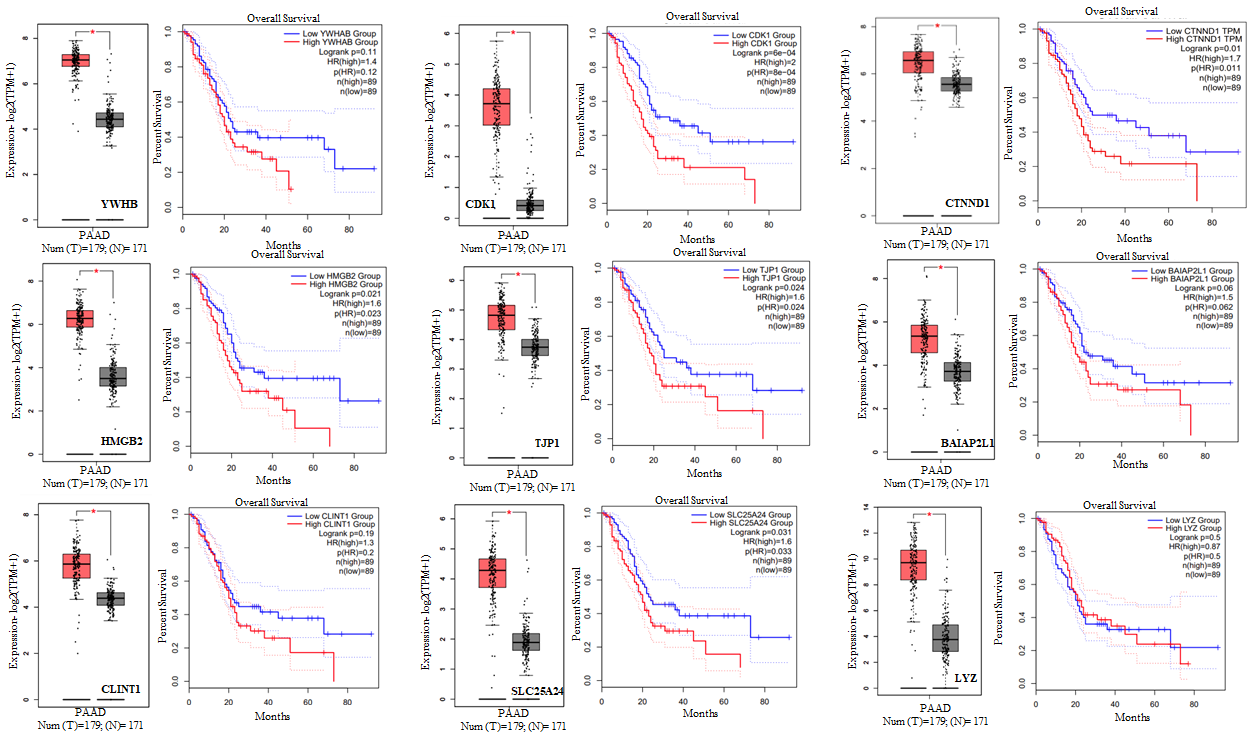


**Supplementary figure 4:** MUC13 associated proteins and their status in the term of expression of genes in pancreatic cancer condition and overall survival of patients if the particular gene is overexpressed.
